# Standardization of Data for Clinical Use and Research in Spinal Cord Injury

**DOI:** 10.3390/brainsci6030029

**Published:** 2016-08-12

**Authors:** Fin Biering-Sørensen, Vanessa K. Noonan

**Affiliations:** 1 Clinic for Spinal Cord Injuries, Rigshospitalet (2081), University of Copenhagen, Copenhagen DK-2100, Denmark; 2 Rick Hansen Institute, Vancouver, BC V5Z 1M9, Canada; vnoonan@rickhanseninstitute.org

**Keywords:** spinal cord injury, standardization, data collection, international data sets, common data elements, electronic medical record

## Abstract

Increased survival after spinal cord injury (SCI) worldwide has enhanced the need for quality data that can be compared and shared between centers, countries, as well as across research studies, to better understand how best to prevent and treat SCI. Such data should be standardized and be able to be uniformly collected at any SCI center or within any SCI study. Standardization will make it possible to collect information from larger SCI populations for multi-center research studies. With this aim, the international SCI community has obtained consensus regarding the best available data and measures for use in SCI clinical practice and research. Reporting of SCI data is likewise standardized. Data elements are continuously updated and developed using an open and transparent process. There are ongoing internal, as well as external review processes, where all interested parties are encouraged to participate. The purpose of this review paper is to provide an overview of the initiatives to standardize data including the International Spinal Cord Society’s International SCI Data Sets and the National Institutes of Health, National Institute of Neurological Disorders and Stroke Common Data Elements Project within SCI and discuss future opportunities.

## 1. Introduction

Survival following spinal cord injury (SCI) has increased remarkably over the last 50 years and a substantial proportion of individuals with SCI worldwide are expected to live for many years with a reasonable quality of life [1,2,3,4]. As a result, there is an increasing need for data pertaining to SCI. In addition, the incidence and prevalence of individuals with SCI is relatively low, therefore it is important to be able to use data from multiple centers and countries to obtain evidence on how to best prevent and treat SCI.

Such data should be standardized and be able to be collected at any SCI center worldwide. Standardization will facilitate comparisons regarding SCI lesions, treatments, and outcomes between patients, centers and countries. Standardized data may also be used as checklists to assist with the observation and treatment of individuals with SCI and form the basis of a (electronic) medical record to be used in SCI centers internationally.

These standardized data will make it possible to collect information from larger SCI populations to inform prevention programs worldwide, as well as facilitate quality improvement initiatives and multi-center research studies (e.g., new devices or medicine for SCI individuals). For the reasons above, an increasing number of regions and countries are establishing SCI registries, databases and electronic medical records including structured information regarding SCI. There is an interest internationally to establish standardized measures/instruments and data elements, which can be used to assess and compare services and outcomes for SCI. When developing new or upgrading existing SCI registries/databases, the possibility of using standardized measures or data elements is essential to compare, as well as collate information between and from various SCI centers.

The first attempt to standardize reporting in the SCI community was the neurological/functional classification of individuals with SCI, called the “Frankel classification”. It divided SCI individuals into grades A, B, C, D, and E according to the motor and sensory completeness of their SCI, as well as the usefulness of their motor-function [5]. The classification was further developed by the American Spinal Injury Association (ASIA) in 1982 and later, in collaboration with the International Spinal Cord Society (ISCoS), into the internationally recognized and currently endorsed standard, the ASIA/ISCoS International Standards for Neurological Classification of Spinal Cord Injury (ISNCSCI); it is used worldwide and the seventh and latest revision was made in 2011 [6,7,8]. In addition, an on-line educational program called InSTeP (International Standards Training E Program) and a classification algorithm for the ISNCSCI was created and can be downloaded free of charge [9,10,11].

To obtain the same worldwide success as the ISNCSCI, the developed measures and data must be simple and relevant to both clinicians and researchers, depending on the context where they will be used. Generally, if clinically relevant, these measures and data elements will also be appropriate for research. To ensure worldwide dissemination and uptake it is likewise imperative that they are easily retrieved, available for use without restrictions and free of charge.

With this aim, the international SCI community has been working to establish consensus regarding the best available data and tools for use in clinical practice and research related to SCI for over a decade [12,13,14,15,16,17,18,19,20,21,22,23]. The purpose of this review is to provide an overview of the initiatives undertaken to standardize data in SCI as part of the International SCI Data Sets and the National Institutes of Health (NIH) National Institute of Neurological Disorders and Stroke (NINDS) Common Data Elements (CDEs) Project and discuss future opportunities.

## 2. International Spinal Cord Injury Data Sets

The roadmap for creating the International SCI Data Sets started at an international workshop on 2 May 2002, prior to the combined meeting of ASIA and ISCoS in Vancouver, Canada, where individuals with expertise in the collection and analysis of SCI data met. At this workshop a process for selecting data elements to include in the International SCI Data Sets was developed [12]. The intent was for the data elements to be collected at the same time as the history and physical examination, or as soon as possible to avoid any recall bias and ensure data quality [12].

### 2.1. Structure and Terminology

**Figure 1:**
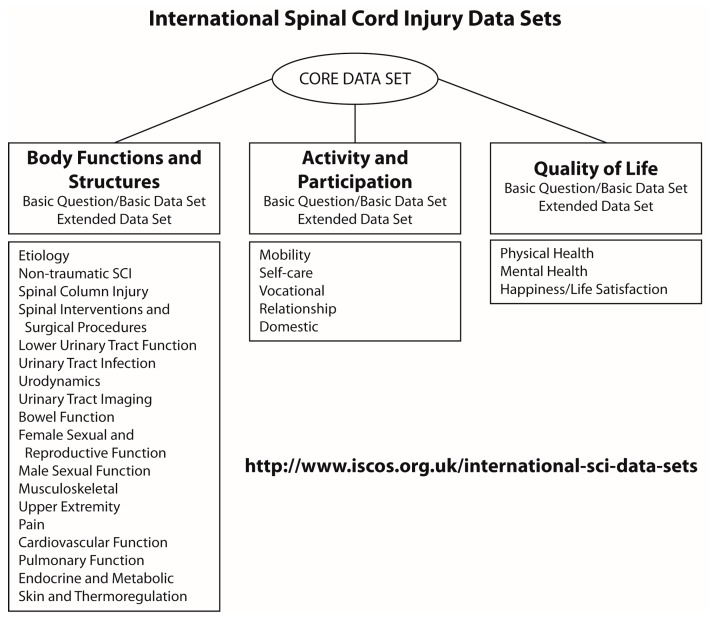
Structure of the International Spinal Cord Injury Data Sets. Modified with permission from Biering-Sørensen, F., Spinal Cord, published by Nature Publishing Group, 2006 [12].

The overall structure and terminology for the International SCI Data Sets was aligned to the International Classification of Function, Disability and Health (ICF) endorsed by World Health Organization [24]. The ICF is an internationally accepted classification of health and health-related states, therefore it was considered to be a useful conceptual framework for the International SCI Data Sets related to consequences of SCI (Figure 1). In the ICF, a person’s functioning or disability is conceived as a dynamic interaction between health conditions, environmental and personal factors.

#### 2.1.1. International SCI Core Data Set

The International SCI Core Data Set is the recommended minimal data set that is collected for all individuals with a new SCI during their initial in-patient period. These data should be included as a descriptive table in most publications that include SCI participants. The International SCI Core Data Set consists of variables regarding basic demographic characteristics, dates of admission and discharge from initial acute and rehabilitation care, cause of injury, place of discharge, presence of bony vertebral injury and associated injuries, occurrence of spinal surgery, and measures of ventilator and neurological status [25].

#### 2.1.2. Basic Questions

These are questions, which with an affirmative answer, it is recommended that a specific International SCI Data Set with detailed information on the particular topic is used. Such questions were not needed for all data sets.

Examples of basic questions:

- Bony vertebral injury: Yes/No/Unknown (from the International SCI Core Data Set) → International SCI Spinal Column Injury Basic Data Set [26]
- Spinal surgery: Yes/No/Unknown (from the International SCI Core Data Set) → International SCI Spinal Interventions and Surgical Procedures Basic Data Set [27]
- Etiology of lesion: Sports/Assault/Transport/Fall/Other traumatic/Non-traumatic (from the International SCI Core Data Set) → Etiology module (SCI version of International Classification of External Causes of Injury (ICECI) [28,29] and the International SCI Data Sets for Non-Traumatic SCI [30]
- Pain: Have you had any pain during the last seven days including today: Yes/No → International SCI Pain Basic Data Set [31,32]

#### 2.1.3. International SCI Basic Data Sets

These data sets include the minimal number of data elements, including a Basic Question for some data sets, which together should be collected in daily clinical practice for a particular topic. Therefore, a combination of the 19 International SCI Basic Data Sets (see Table 1) and the International SCI Core Data Set can, together with the ISNCSCI, form the basis for a structured electronic medical record in centers worldwide caring for persons with SCI.

#### 2.1.4. International SCI Extended Data Sets

These extended data sets are more detailed and optional for a topic, but are recommended for specific research studies within the particular area. Thus far, the International SCI Extended Data Sets include:

- ICECI (International Classification of External Causes of Injury) for SCI [28]
- International SCI Non-traumatic Spinal Cord Injury Data Set [30]
- International SCI Bowel Function Extended Data Set [51]
- International SCI Pain Extended Data Set [52]
- International SCI Endocrine and Metabolic Extended Data Set [53]

Several others are in development.

### 2.2. Development of International Spinal Cord Injury Data Sets

Initially, a joint ASIA and ISCoS Executive Committee for International SCI Standards and Data Sets was established as a steering committee to oversee the specific working groups (WGs) that developed new International SCI Data Sets. This committee structure was in place until late 2014, when it was replaced by the International SCI Data Sets Committee under the ISCoS Scientific Committee, and with support from ASIA. International and larger national organizations and societies within related fields (e.g., neurosurgery, orthopaedic surgery, urology, rehabilitation, and psychology) were invited to appoint members to join the review process to enhance the content of the International SCI Data Sets. For specific topics, relevant organizations may be asked to appoint individuals to the WGs, e.g., the International Association for the Study of Pain to the International SCI Pain Data Set group. In addition, anyone else who is interested can be added to the list of reviewers. All International SCI Data Sets are reviewed by relevant committees and interested organizations and individuals. Then they are revised accordingly, in an iterative process and finally approved and endorsed by interested organizations.

#### 2.2.1. Process for approval and publication of International SCI Data Sets

The process includes the following steps:

(1) The particular SCI Data Set WG group finalizes the topic specific SCI Data Set.
(2) The International SCI Data Sets Committee reviews the SCI Data Set.
(3) Comments from the Committee are discussed in the particular SCI Data Set WG and a response is prepared and the SCI Data Set may be adjusted.
(4) ISCoS Scientific and Executive Committees and ASIA Board review the SCI Data Set.
(5) Comments from the Committees/Board are discussed in the particular SCI Data Set WG and a response is prepared and possible adjustments of the SCI Data Set are made.
(6) Relevant and interested (International) Organizations and Societies—around 40—and individuals are invited to review the SCI Data Set. In addition, the SCI Data Set is posted on the ASIA and ISCoS websites for at least one month for comments.
(7) Comments are discussed in the particular SCI Data Set WG and a response is prepared and adjustments to the SCI Data Set are made if necessary.
(8) ISCoS Scientific and Executive Committees and ASIA Board review the SCI Data Set for final approval.
(9) The SCI Data Set is then further reviewed by the NIH NINDS CDE Project team to develop standardized variable names and a database structure for each of the International SCI Data Sets [54,55].
(10) The SCI Data Set is posted on the ISCoS and NIH NINDS CDE websites [33,56] and published in the journal Spinal Cord.
(11) Endorsement of the SCI Data Set by relevant (International) Organizations and Societies.

### 2.3. Reliability and Validation

As described previously, the International SCI Data Sets are developed using an iterative consensus process using the best available evidence. To further improve the data sets, reliability and validity studies are encouraged [57]. Gradually more of the data sets are being evaluated in these types of studies [58,59,60,61,62,63,64].

### 2.4. Translations

ISCoS welcomes translations of the International SCI Data Sets, and these should preferably include the Syllabus published on the ISCoS website [33]. The translation is not a word-for-word translation, but instead ensures conceptual equivalence. For each translation “the name, role and background of everyone involved in the translation process” must be stated, as well as the date and a version number [57]. The translation has to be performed by individuals with expertise in the SCI specific topic that the International SCI Data Set covers. The translation may be performed with forward- and back-translation. Another approach is that the translation from English into the target language is critically reviewed by one or more experts, confirming that the translation conveys the concepts included in the original English version [57]. All translated data sets that are performed in accordance with these guidelines are then uploaded on the ISCoS website [33]. To date, several official Chinese translations are available.

### 2.5. Reporting

Uniformity in reporting of SCI data is warranted in order to facilitate comparison between studies and guidelines [65]. This reporting includes variables such as age, time since SCI, calendar year and severity of the SCI. These recommendations were based on the experience reporting data from a large SCI database and also by individuals who assisted with the development of the International SCI Data Sets (Table 2) [65]. The journal Spinal Cord supports these recommendations in the “Instructions for Authors”, to ensure submitting authors report variables in a uniform manner.

## 3. NINDS Common Data Elements for Spinal Cord Injury Clinical Research

The NIH NINDS CDE Project was initiated in 2006 with the aim of developing CDEs, data definitions, case report forms (CRFs) and guidelines relevant to clinical research in neurological diseases [66]. The NIH NINDS CDE Project for SCI began in 2012.

### 3.1. Terminology

The SCI CDEs and measures are classified according to the following definitions [22].

#### 3.1.1. Core CDE

A Core CDE is a data element, which is strongly encouraged to be used in all SCI studies. The aim is to provide consistency across studies regarding basic participant information. These also include CDEs that the NINDS has defined as “Core for All Neurological Diseases”.

#### 3.1.2. Supplemental CDE

A Supplemental CDE is a data element or measure, which is recommended for a significant number of SCI studies, but may not be relevant for all studies depending on the study design and the type of research being conducted. The Supplemental CDEs most highly recommended for specific types of studies are designated as “Supplemental/Highly Recommended”.

#### 3.1.3. Exploratory CDE

An Exploratory CDE is a data element or measure, which requires further validation before reaching a consensus and recommendation for inclusion as a Supplemental CDE.

### 3.2. Development of NINDS Common Data Elements for Spinal Cord Injury

NINDS, ISCoS, and ASIA established an organizing committee, with five to seven SCI experts representing clinicians, clinical researchers, including clinical trials, industry, private and public funding organizations to be on the WGs for the following domains:

(1) Demographics;
(2) Care;
(3) Neurological outcomes;
(4) Functional outcomes;
(5) Pain, including Psychological outcomes;
(6) Participation and Quality of Life;
(7) Electro diagnostics;
(8) Imaging.

Within each of the above domains, the WG members reviewed the existing data elements and measures and identified the most important ones that should be used for clinical SCI studies. The NIH NINDS CDE Project team supported the WGs by identifying relevant measures and data elements [66], including the Patient Reported Outcome Measurement Information System (PROMIS) measures [22]. After the review, the WGs recommended which data elements and measures should be included, developed guidelines outlining how they should be used in SCI studies and created CRFs for selected CDEs. Due to differences in the availability of CDEs and measures for the various WG domains, the process differed among the WGs. The WGs for Demographics and Care initially reviewed CDEs for other health conditions (e.g., stroke), other SCI clinical studies and SCI registries and selected CDEs and tools that were most appropriate for SCI studies. The WGs for Neurological and Functional outcomes critically reviewed the existing SCI assessment measures and their psychometric properties (e.g., reliability and validity) and made recommendations for their use in SCI studies. The WGs for Participation and Quality of Life and Pain included the Psychological outcomes, selected measures used in SCI, as well as from other fields for consideration in SCI. The Electro diagnostics and Imaging WGs were required to develop completely new CDEs and measures since there was nothing available in these areas [22].

The NIH NINDS SCI CDE Project includes individuals involved with the clinically-directed International SCI Data Sets and uses the same standard variable names and common database structure. Furthermore, cross referencing with the ISCoS International SCI Data Sets ensures a common language across the full spectrum of clinical research studies worldwide.

In total there are 1150 data elements and measures with definitions, CRFs and guidelines included in the NINDS CDEs for SCI. These include seven Core CDEs required for all NINDS CDE data sets, two Core CDEs specific for SCI, i.e., the date and etiology of injury, and one Core measure, the ISNCSCI [7]. There are eight Highly Recommended Supplemental measures for clinical SCI research and these measures include several individual CDEs. Otherwise the CDEs and measures were recommended as either Supplemental or Exploratory [22].

#### 3.2.1. Process for Review and Publication of the NINDS CDEs for Spinal Cord Injury Clinical Research

After each WG selected the CDEs and measures and provided definitions, CRFs, and guidelines, these were then reviewed internally by all the WG members and the NIH NINDS CDE Project team. When the appropriate adjustments were made, the document was approved for external review. This was accomplished by publishing the entire document on the NIH NINDS CDE website, as well as the ISCoS website. At the same time, e-mails were sent to SCI organizations and research institutions, foundations, industry and SCI consumers, as well as individuals with interest in SCI and research, and all ISCoS members. The suggestions and comments were provided to the appropriate WGs and revisions were made accordingly.

The finalized Version 1.0 of the NINDS SCI CDEs was published 30 August 2014 on the NIH NINDS CDE website [67] and can also be found through a link on the ISCoS website [33].

## 4. Challenges

When considering standardizing data collection it is always important to consider the purpose and ensure it is appropriate. The recommendations for uniformity in data collection should not overshadow, for example, other objectives of a research project where different measures or data elements may be more appropriate. This may include surveillance studies using diagnostic codes from administrative data, as well as studies including several disability groups, in addition to SCI. Longitudinal studies that were initiated before the development of the International SCI Data Sets and CDEs may have to continue with other data sets or elements, but should be advised to include the recommended elements when they are available and relevant. Furthermore many data fields do not yet have international standardized data elements at this time.

Cultural differences between countries may be greater for some than for others, which imply that the use of some data elements may be more difficult to implement globally. However, the WGs have strived to make the variables/elements as universal as possible, not least by having individuals from various areas of the world as participants in the WGs or as reviewers. The International SCI Data Sets are currently being used within the international SCI community in an increasing number of registries worldwide, including all eight SCI centers in the Netherlands, the Nordic SCI Registry, the Rick Hansen SCI Registry in Canada, in Victoria, Australia (and being extended nationally), and in a prevention registry covering India, Nepal, Sri Lanka, Bangladesh, Malaysia, and Thailand. Translations for several of the International SCI Data Sets into Dutch, Spanish, Portuguese, Italian, French, Korean, and the Nordic languages are forthcoming. As mentioned previously, several official Chinese translations are currently available.

Regarding standardizing reporting, it is important to be aware that in certain circumstances there may be reasons not to follow the recommended framework. For example, if studies have a limited number of participants it may not be possible to divide data in accordance with the recommendations. It is advisable, however, to adhere as much as possible to the recommendations to facilitate comparisons and to increase the availability of SCI information for future research.

## 5. Conclusions and Future Development

The worldwide acceptance of the ISNCSCI [6], the ISCoS International SCI Data Sets [12] and the recent NIH NINDS CDEs Project [22] has paved the road for sharing clinical and research data between SCI centers and studies. The data elements and measures are important resources to facilitate standardized data collection (e.g., in electronic medical records) within SCI centers worldwide and likewise when developing protocols for SCI studies.

It is also important to realize that all data elements and measures suggested are based on the current knowledge and experience available in the field of SCI. Therefore, as new evidence is produced the variables, items and measures will be updated. This process for changes, additions and removal of data elements and measures will be as public and transparent as possible. There will be an ongoing internal as well as an external review process where all interested parties are welcome to participate. It will also be important to evaluate the uptake of the International SCI Data Sets in both clinical practice and research. Lessons learned from these initiatives can inform other health conditions embarking on data standards for their field.

## Figures and Tables

**Table 1:** The 19 International Spinal Cord Injury (SCI) Basic Data Sets [33].

International SCI Non-traumatic Spinal Cord Injury Data Sets [30]
International SCI Spinal Column Injury Basic Data Set [26]
International SCI Spinal Interventions and Surgical Procedures Basic Data Set [27]
International SCI Lower Urinary Tract Function Basic Data Set [34]
International SCI Urinary Tract Infection Basic Data Set [35]
International SCI Urodynamic Basic Data Set [36]
International SCI Urinary Tract Imaging Basic Data Set [37]
International SCI Bowel Function Basic Data Set [38]
International SCI Female Sexual and Reproductive Function Basic Data Set [39]
International SCI Male Sexual Function Basic Data Set [40]
International SCI Musculoskeletal Basic Data Set [41]
International SCI Upper Extremity Basic Data Set [42,43]
International SCI Pain Basic Data Set [31,32]
International SCI Cardiovascular Function Basic Data Set [44]
International SCI Pulmonary Function Basic Data Set [45]
International SCI Endocrine and Metabolic Function Basic Data Set [46,47]
International SCI Skin and Thermoregulation Basic Data Set [48]
International SCI Activity and Participation Basic Data Set [49]
International SCI Quality of Life Basic Data Set [50]

**Table 2:** Standardization of reporting of data on spinal cord injuries [65].

**Age at SCI:**	**Mean, Standard Deviation, Median, Range**
When grouped:	15-year increments: 0–15, 16–30, 31–45, 46–60, 61–75 and 76+ years
For pediatric SCI:	0–5, 6–12, 13–15, 16–21 years
**Time since SCI:**	**Mean, standard deviation, median, range**
When grouped:	0–1 year, 1–5, 6–10, 11–15 years, and 5-year increments thereafter
**Calendar time:**	**Years during which the study is conducted**
When grouped:	By either 5 or 10-year increments with years ending in 4 or 9
**Length of stay:**	**Mean, standard deviation, median, range**
Severity of SCI:	C1–4 AIS A, B or C
	C5–8 AIS A, B or C
	T1–S5 AIS A, B, or C
	AIS D at any injury level
	Ventilator dependent (for all injuries levels and AIS grades)

SCI, Spinal Cord Injury; C1-4 and C5-8 correspond to the cervical spinal cord levels of SCI; T1-S5 correspond to the thoracic, lumbar and sacral spinal cord levels of SCI; AIS, American Spinal Injury Association Impairment Scale grade, the grades are A, B, C, D, and E [7].
